# A Short History of Six Cases of Placenta Prævia

**Published:** 1871-07

**Authors:** E. R. Willard

**Affiliations:** Wilmington, Ill.


### CHICAGO MEDICAL EXAMINER:

N. S. DAVIS, M.D., EDITOR.

F. H. DAVIS, M.D., ASSISTANT-EDITOR.

VOL. XII. JULY, 1871. NO. 7.

### Original Contributions.

### A SHORT HISTORY OF SIX CASES OF PLACENTA PRÆVIA.

BY E. R. WILLARD, M.D., of Wilmington, Ill.

CASE I.—In August, 1853, Mrs. R——, aged about 30, multipara, of nervous temperament, quite hysterical, and in poor health, was taken with slight hemorrhage about three weeks before full time. I was sent for; found her still flowing some, but without pain; learned that she had been having what she called a show at different times for six weeks previous. As the hemorrhage was slight, and there seemed to be no necessity for active interference, I ordered quinine and iron to relieve the debilitated anemic condition of the system, with strict orders that she should keep the recumbent position for several days. From this the hemorrhage ceased, and the general health improved for ten days, when she was again taken with hemorrhage, which was more profuse than on the former occasion, but not sufficient to excite alarm, and subsided spontaneously. About the end of pregnancy I was again summoned in great haste; found her flowing so profusely as to have resulted in partial syncope. Upon making an examination per vaginum I found the os dilated to the size of half-a-dollar, and my worst fears realized by finding the placenta in the interval. I immediately introduced a tampon and held it up with some considerable force, ordering at the same time counter pressure upon the abdomen by two women, who with the flat hands firmly pressed the uterus and contents well down. This procedure was continued some three-quarters of an hour, when during a pain the tampon was removed, the placenta detached upon the left side, which seemed the thinnest, and the membrane ruptured. Continuous pressure was still maintained upon the abdomen, the tampon again introduced, after complete evacuation of the liquor amnii, and held firmly up as before. By this time the ergot, which had been given in the form of an infusion in the proportion of an ounce to a pint of water, began to make a decided impression upon the uterus, the contractions becoming almost continuous. As the child's head, which was presenting, soon became engaged, I removed the tampon, but persisted in the downward pressure, and had the satisfaction, inside of an hour from the time of rupturing the membranes, of delivering the child, which was finally resuscitated after much careful manipulation. The placenta came away in five minutes.

Although neither the delivery of the child or placenta was accompanied with any considerable amount of flooding, still the patient was so bloodless, from previous hemorrhages before my arrival, as to make it necessary to place the head considerably below the rest of the body to prevent syncope.

I remained with the patient a few hours to insure perfect reaction, or sufficient, as I thought, to relieve apprehension of further danger, and then rode into the country. I was gone some three hours. Upon returning, to my surprise, I found the patient in articulo-mortis. She only made two or three ineffectual efforts at respiration after my arrival. I attributed the unfortunate termination of this case to the interference of two old ladies, who, in my absence, persisted in elevating the woman's head a foot or more above the rest of the body, thereby causing fatal syncope. This I inferred from finding the patient in that condition. I immediately lowered the head and applied stimulants and friction, all to no purpose. Upon examination the uterus was found to be firmly contracted, without any signs of hemorrhage more than a slight oozing since the birth of the child. The fatal result in this case produced a decided impression upon my mind at the time, as I was most certain that the patient would recover after the completion of the accouchement.

CASE II.—In August, 1854, I was summoned to see Mrs. B——, in consultation with Dr. Jones, of Channehon. The messenger stated that he feared the woman would be dead before my arrival unless I made great haste. I found the patient about 28 years of age, nervous temperament, multipara, not much advanced beyond the sixth month of gestation, in a very critical state. Her countenance was pinched and anxious, pulse hardly perceptible, breathing short and quick, and the body bathed in a profuse cold perspiration. I learned from the friends that she had been affected by hemorrhage that recurred every week without specific cause for three weeks past, and that she had been having some pain since the flow commenced this time. Upon examination I found the bed saturated with blood, the vagina distended by a large clot, and hemorrhage going on actively. The cervex dilated to the size of an American quarter, through which the placenta could be plainly felt, covering the entire dilated orifice, and subsequently proved to be centrally attached.

I recommended the immediate separation of the placenta and free administration of ergot, as the best means in my judgment calculated to relieve the urgent symptoms in the case. With the request of the attending physicians I introduced my hand into the vagina, and with the fingers in the uterus detached the placenta without rupturing the membranes. The hemorrhage immediately ceased. Ergot was ordered in full doses every twenty minutes, together with wine and beef essence, and the patient well surrounded with artificial heat. In an hour, the pains began to increase in frequency and force, and in three hours labor was terminated, the uterus expelling the fetus, placenta, and membranes entire. After agreeing upon the treatment to be adopted to bring on reaction, I left the patient in charge of the attending physician, with the most sanguine expectation that she would recover.

These anticipations were not fulfilled, as I afterwards learned from the friends that the patient died in about six hours from my departure. I have always been of the opinion that the fatal termination of the case was the result of post partum hemorrhage, that might possibly have been stayed, had due diligence and care been used in the subsequent management of the case.

Case III.—On the morning of January 12th, 1860, Dr. E. H. Strong sent for me to see a patient of his in her sixth month of gestation. Upon arriving found Mrs. C——, a strong, muscular, multiparious woman, who, without premonitory hemorrhage, was taken ill the evening before, after lifting some heavy articles, with alarming flooding, and now lay pale and almost pulseless. Dr. Strong was sent for in the night shortly after the flooding commenced, and immediately introduced the tampon, which for a time controlled the flow, but towards morning slight uterine contractions came on, producing periodic hemorrhage of considerable violence, which induced him to send for me.

Upon removing the tampon and making a vaginal exploration, I found the os slightly dilated, just sufficient to introduce the index-finger. As the hemorrhage was going on actively I passed the finger through the cervex, and distinctly felt the placenta, which subsequently proved to be nearly centrally attached, membranes entire. As the patient was so completely exhausted that further loss of blood would doubtless result in the fatal termination of the case, I proposed the entire separation of the placenta as the best means under the circumstances calculated to control the flow, and give time for the action of ergot and stimulants. This being agreed upon, I introduced the hand, and with the fingers in the uterus easily detached the placenta, without rupturing the membranes. The tampon was again introduced and the ergot given in full doses every twenty minutes. The hemorrhage ceased almost immediately, with the exception of slight oozing, and in two hours the whole contents of the uterus was expelled, child, placenta, and membranes entire. I watched the patient carefully during the entire time, never leaving the bedside, and as the case terminated so favorably, it left no doubt upon my mind in regard to the mode of procedure in this case being correct. The mother made a good recovery.

CASE IV.—*Jan. 26th, 1871.*—Dr. Abbott called at my office, and requested me to go with him to see Mrs. B——, three miles distant. During the ride he gave me the following history: Two weeks previous the patient, a multipara, of bilious sanguine temperament, in good health, was affected, without assignable cause, by hemorrhage of considerable severity, which ceased spontaneously before his arrival. She was directed to maintain the recumbent position as much as possible, and avoid all exertion more than was absolutely necessary. About a week from this, she was again taken in the same way, but less severe; the flow also ceased spontaneously, same as on the former occasion. This morning he found the woman flowing quite profusely, accompanied with some pain. After an hour and a-half's attendance, and the hemorrhage had become alarming, he was induced to call me in.

I found the patient cold, quite faint, pale, and almost pulseless, with some nausea and ineffectual efforts at vomiting. Said it was too dark to tell who I was when I spoke to her, although the room was quite light at the time. Examination revealed a large clot distending the vagina; the os dilated to an inch in diameter, and completely dilatable, through which the placenta could be distinctly felt blocking up the entire cervex. The left edge subsequently proved to have crossed over, and overlapped the os in its present dilated condition about an inch. Upon consultation, we concluded to immediately turn the child and deliver the woman—as the soft parts were very much relaxed, the os dilatable, and the liquor amnii present—hoping thereby to save both mother and child. A full dose of ergot was given, and pedalic version performed without delay. The feet being secured, firm pressure was maintained upon the abdomen, and the delivery completed in less than twenty minutes. The placenta soon followed.

After resorting to artificial respiration for some ten minutes, together with warm baths, the child was finally resuscitated. The mother lay in a critical condition for several hours, but subsequently rallied, and made a good recovery.

Case V.—On December 15th, 1868, I was summoned to Mrs. W——, in consultation with Dr. W. R. Fox, eight miles distant. The patient, a multipara, of nervous bilious temperament—supposed to be in the sixth month of gestation—had for several weeks previous been suffering from uterine hemorrhage, more or less severe, without specific cause. She had lost a large quantity of blood on the day of the visit; her face exhibiting a somewhat blanched and cadaveric appearance; pulse weak and very much accelerated; and so much exhausted as to become quite dizzy upon lifting her head from the pillow, with great depression of spirits.

Upon removing the tampon previously introduced by Dr. Fox, and making a vaginal examination, the os uteri was found dilated sufficient to admit the index-finger, the edge thin and yielding, through which the placenta could be felt overlapping the whole of the dilated orifice. Slight uterine contractions having occurred at intervals of an hour during the greater part of the day, it was agreed that premature delivery would promise best for the safety of the patient. Accordingly, the placenta was partially detached, the tampon reintroduced without rupturing the membranes, and ergot freely given. Expulsive pains soon came on, and in a few hours the entire contents of the uterus were expelled, without further material loss of blood. Examination showed the placenta to have overlapped the os about an inch. The patient made a good recovery.

Case VI.—*April 10th, 1871.*—Mrs. C——, the same as reported in Case III., over 40 years of age, and mother of 15 children, sent for me in great haste. When I saw the patient about 5 P.M., I found the bed so completely saturated as to have run through, and formed a large pool upon the floor. Pulse frequent and small, with nausea, vomiting, and profuse perspiration. There was no premonitory hemorrhage in this case up to this time, and she only wanted about two weeks of completing her present gestation. I learned that she was taken, two hours previous to my visit, with profuse hemorrhage, without either pain or other assignable cause. Upon examination, I found the edge of the placenta crossing the cervex, which was dilated sufficient to easily admit the index-finger. I immediately introduced the tampon, and held it firmly up against the os uteri, accompanied by counter pressure by assistance over the uterus; at the same time, gave full doses of ergot every 20 minutes, until strong uterine contractions came on.

At 6 P.M., the pains having become quite severe, I ruptured the membranes and evacuated the liquor amnii, hoping thereby that the placenta would become compressed by the descent of the head to such an extent as to control the hemorrhage. My anxiety for the success of this plan was extremely great, as the pulsations of the child's heart could be distinctly heard, and I was in hopes that it might be delivered without resorting to version, thereby giving the child the greatest possible chance of safety.

These anticipations were fully realised; as, shortly after rupturing the membranes, the uterine contractions became almost continuous, and the hemorrhage was diminished so much as to require but little interference. I had the firm pressure continued over the uterus until the head had entered the superior strait, and in a little over three hours from the time of my arrival at the house, the patient was delivered of a living male child. The mother made a good recovery.

REMARKS.—It is generally conceded by all medical writers that the attachment of the placenta over the os internum is as certainly fatal to the child-bearing woman as the most malignant epidemic of yellow fever or cholera—the mortality being as one to three in all cases having this complication. Although elaborate articles have been written by all our best obstetricians, both on this and the Eastern Continents—each differing more or less from the other in their peculiar management of these cases—still the mortality does not seem to have been materially changed: this no doubt is in consequence of the patients not being seen by the practitioner until the hemorrhage has become so great as to be irremediable. I am of the opinion that very much depends upon the judgment of the physician in attendance—always bearing in mind the two indications presented, of diminishing the hemorrhage and completing the labor as soon as possible. To fulfil the first of these indications, I have found the tampon held firmly against the os, assisted by pressure upon the abdomen, to be the most effectual. Should the flooding be so profuse as to endanger the life of the patient, and this before her seventh month of gestation, I would recommend the complete separation of the placenta, without extracting it from the uterus (as advocated by Prof. Simpson) or rupturing the membranes.

And for the second indication, ergot should be given in full and repeated doses. The os uteri, if not quite elastic and dilatable, should be artificially dilated by means of the internal caoutchouc dilator. I would not recommend the early evacuation of the liquor amnii unless gestation was nearly completed, and the os sufficiently dilatable to ensure the rapid descent of the fetus. Even then, the possible necessity of version should be taken into consideration, as there would be little danger from internal hemorrhage so long as the uterus remained full, and the requisite amount of continuous pressure was made upon the abdomen. I have found the application of a sheet—the same as for *parecentesis abdominis*—to answer admirably for that purpose. The external flow can also be more effectually controlled by continuous firm pressure with a proper fitting tampon. In absence of anything better, I have found a small-sized glass speculum, properly padded, and inserted wrong end up, to answer a good purpose. In one case, a castor-oil bottle, padded as above, and inserted bottom up, did good service. This procedure necessitates the constant personal attention of the physician until the accouchement is completed.

